# The DNA transporter ComEC has metal‐dependent nuclease activity that is important for natural transformation

**DOI:** 10.1111/mmi.14720

**Published:** 2021-06-04

**Authors:** Augustinas Silale, Susan M. Lea, Ben C. Berks

**Affiliations:** ^1^ Department of Biochemistry University of Oxford Oxford UK; ^2^ Sir William Dunn School of Pathology University of Oxford Oxford UK; ^3^ Present address: Biosciences Institute Newcastle University Newcastle Upon Tyne UK; ^4^ Present address: Center for Structural Biology, Center for Cancer Research National Cancer Institute Frederick MD USA

**Keywords:** competence, DNA transporter, Gram‐positive bacteria, natural transformation, nuclease

## Abstract

In the process of natural transformation bacteria import extracellular DNA molecules for integration into their genome. One strand of the incoming DNA molecule is degraded, whereas the remaining strand is transported across the cytoplasmic membrane. The DNA transport channel is provided by the protein ComEC. Many ComEC proteins have an extracellular C‐terminal domain (CTD) with homology to the metallo‐β‐lactamase fold. Here we show that this CTD binds Mn^2+^ ions and exhibits Mn^2+^‐dependent phosphodiesterase and nuclease activities. Inactivation of the enzymatic activity of the CTD severely inhibits natural transformation in *Bacillus* *subtilis*. These data suggest that the ComEC CTD is a nuclease responsible for degrading the nontransforming DNA strand during natural transformation and that this process is important for efficient DNA import.

## Graphical abstract

During natural transformation in bacteria one DNA strand is transported into the cytoplasm via the membrane protein ComEC while the other strand is degraded. Here we show that the C‐terminal domain of ComEC has a nuclease activity that is important for natural transformation in *B*. *subtilis*. Thus, this domain is likely to be responsible for digesting the non‐transforming DNA strand.

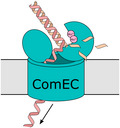

## 1 INTRODUCTION

Natural transformation is a mechanism of horizontal gene transfer in which bacteria take up DNA from their environment and integrate it into their genome (Dubnau & Blokesch, 2019; Johnston et al., 2014). Transformation allows bacteria to acquire adaptively useful genes, to operate a distributed gene pool, and to remove parasitic mobile genetic elements from their genomes (Carvalho et al., 2020; Croucher et al., 2016; Power et al., 2021). In pathogenic bacteria, natural transformation is important for the spread of virulence traits including capsule variation and antibiotic resistance (Chi et al., 2007; Croucher et al., 2011; Domingues et al., 2012; Griffith, 1928). Specific physiological conditions are normally required for expression of the transformation machinery, with those cells developing the ability to import DNA being said to have reached a state of competence. Only about 80 bacterial species have been experimentally shown to be transformable (Johnston et al., 2014). However, homologues of core competence genes are found in most bacterial genomes suggesting that the majority of bacteria undergo transformation in their natural habitats (Carvalho et al., 2020; Pimentel & Zhang, 2018).

DNA uptake during transformation occurs in two stages. In the first step extracellular DNA is transported across the outer membrane (where present) and cell wall. This step is normally mediated by a retractile type IV pilus (Ellison et al., 2018) with subsequent capture of the DNA molecule by the protein ComEA (Gangel et al., 2014; Inamine & Dubnau, 1995; Provvedi & Dubnau, 1999; Seitz et al., 2014), although other mechanisms are known (Brimacombe et al., 2015; Damke et al., 2019; Hofreuter et al., 2001). In the second step, a single strand of the DNA molecule is moved across the cytoplasmic membrane by the protein ComEC (Corbinais et al., 2016; Draskovic & Dubnau, 2005; Inamine & Dubnau, 1995; Seitz & Blokesch, 2013; Stingl et al., 2010). In Gram‐positive bacteria, this movement appears to be assisted by the ATP‐dependent pulling action of the competence‐specific DNA helicase ComFA located at the cytoplasmic side of the membrane (Diallo et al., 2017; Londono‐Vallejo & Dubnau, 1994a, Londono‐Vallejo & Dubnau, 1994b).

All ComEC proteins contain a conserved “competence domain” which is thought to form a transmembrane channel through which the DNA molecule is transported. In most ComEC proteins the competence domain is bracketed by either one or both of two extracellular soluble domains (Pimentel & Zhang, 2018). The N‐terminal soluble domain (NTD) contains an oligonucleotide binding fold, which is likely to associate with the transporting DNA strand (Baker et al., 2016). The water‐soluble C‐terminal domain (CTD) has a metallo‐β‐lactamase (MBL) fold (Baker et al., 2016).

During transformation with double‐stranded DNA only one of the strands is transported into the cytoplasm through ComEC and the other strand is degraded (Dubnau & Blokesch, 2019; Piechowska & Fox, 1971). The reason why the incoming double‐stranded DNA is converted to single‐stranded DNA is not known. However, single‐stranded DNA has been speculated to provide a better substrate for recombination, to reduce susceptibility of the imported DNA to restriction enzymes, and to permit cytoplasmic single‐stranded DNA binding proteins to assist transport through a Brownian ratchet mechanism (Dubnau & Blokesch, 2019). It is also possible that the greater flexibility or smaller cross‐sectional area of single‐stranded DNA is necessary to achieve transport through the ComEC channel. In the Gram‐positive bacterium *Streptococcus* *pneumoniae*, the nontransforming strand is hydrolyzed at the exterior face of the cytoplasmic membrane by the endonuclease EndA (Berge et al., 2002; Mejean & Claverys, 1993; Puyet et al., 1990). Other transformable bacteria lack EndA homologues, and no alternative nucleases involved in degrading the nontransforming strand have been identified, although a nuclease that introduces double‐strand breaks in the incoming DNA (NucA) is found in *Bacillus* *subtilis* (Provvedi et al., 2001). Intriguingly, in a *B*. *subtilis* *comEC* mutant the cell‐associated DNA remains double‐stranded (Provvedi et al., 2001) raising the possibility that in this organism ComEC is itself the nuclease responsible for degrading the nontransforming strand (Inamine & Dubnau, 1995). More specifically, it has been proposed that the nuclease is the MBL‐like CTD of ComEC that is located on the external side of the cytoplasmic membrane (Baker et al., 2016; Dubnau & Blokesch, 2019). However, the proposed nuclease activity of the ComEC CTD has not been experimentally tested.

**FIGURE 1:**
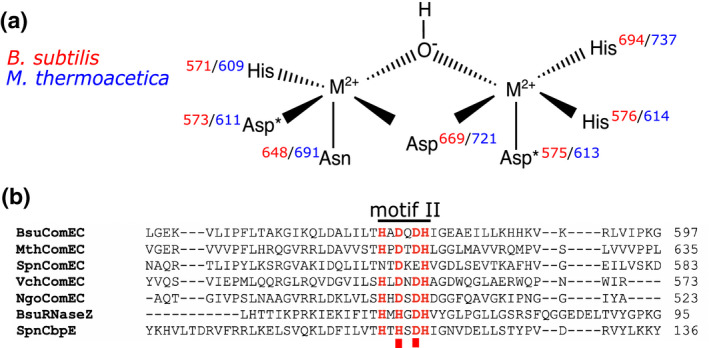
Predicted metal‐ion‐binding residues in the ComEC metallo‐β‐lactamase (MBL) domain. (a) A model for the metal ion (M^2+^) co‐ordination in the ComEC CTD based on structural similarity to the MBL family. Residue numbers are in red for the *Bacillus subtilis* protein and blue for the *Moorella thermoacetica* protein. A metal ion‐bridging hydroxide ion is the likely nucleophile in the hydrolytic reaction catalyzed by the enzyme. *indicates the residues substituted in this study. (b) Alignment of the motif II sequences of ComEC proteins and selected MBLs. Putative or established metal ion–ligating residues are shown in red. The motif positions substituted in this study are marked by red squares. Bsu, *B*. *subtilis*; CbpE, teichoic acid phosphorylesterase; Mth, *M*. *thermoacetica*; Ngo, *Neisseria gonorrhoeae*; Spn, *Streptococcus pneumoniae*; Vch, *Vibrio cholerae*. For a discussion of SpnComEC motif II, see the text

Most MBLs contain two catalytic Zn^2+^ ions that are predominantly co‐ordinated by aspartate and histidine residues provided by five conserved sequence motifs (termed motifs I‐V) (Dominski, 2007; Palzkill, 2013). However, iron‐specific and metal‐promiscuous MBLs have also been reported (Frazao et al., 2000; Schilling et al., 2003). The catalytic activity of MBLs arises from activation of a water molecule bound by the metal ions (Palzkill, 2013; Pettinati et al., 2016). Canonical MBLs hydrolyse the amide bond of the β‐lactam ring in β‐lactam class antibiotics. Other members of the MBL family include nucleases involved in DNA repair and RNA maturation (Dominski, 2007). The suggestion that the ComEC CTD has nuclease activity is, thus, consistent with the known catalytic range of the MBL family. The ComEC CTD conserves most of the metal ligands found in the closest homologue of known structure, the Zn^2+^‐binding teichoic acid phosphorylcholine esterase of *S*. *pneumoniae* (Hermoso et al., 2005), with the exception that an aspartate replaces one of the metal‐co‐ordinating histidine ligands provided by motif II and an asparagine replaces the histidine of motif III (Figure 1a,b).

Here we have investigated the metal‐binding properties and catalytic activity of the ComEC CTD. We find that the CTD binds Mn^2+^ ions and has general phosphodiesterase and nuclease activities. Disruption of the CTD metal binding site abolishes nuclease activity and strongly impairs the ability of ComEC to support transformation in *B*. *subtilis*. These data support the hypothesis that the ComEC CTD is involved in degrading the nontransforming strand of dsDNA and that this activity is important for successful DNA uptake.

## 2 RESULTS

### 2.1 Metal ion binding by the ComEC C‐terminal domain

The MBL‐like CTD of the DNA translocator ComEC is proposed to degrade the nontransforming DNA strand during natural transformation (Baker et al., 2016; Dubnau & Blokesch, 2019). We decided to directly test this hypothesis by isolating the CTD and assessing whether it has metal‐dependent nuclease activity. Note that the full‐length ComEC protein has so far proved refractory to purification (Diallo et al., 2017; Inamine & Dubnau, 1995; Yeh et al., 2003).

**FIGURE 2:**
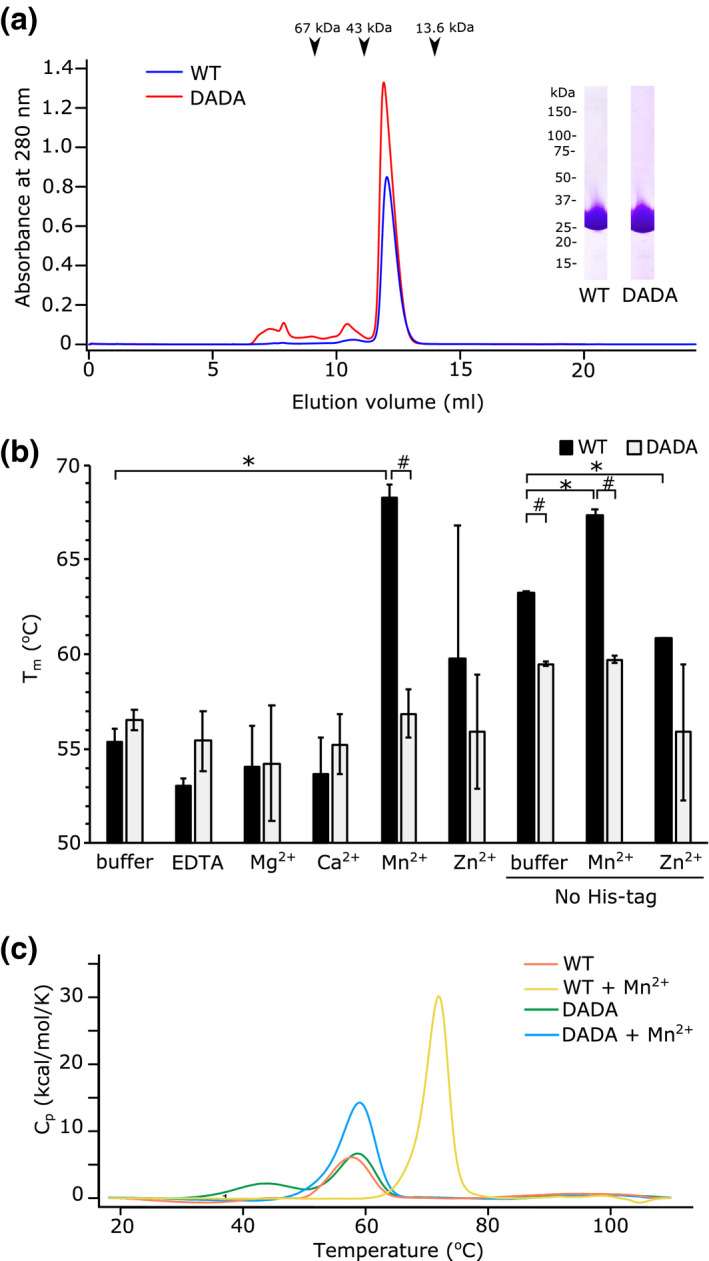
Metal ion binding by the CTD of MthComEC. (a) Size exclusion chromatography on a Superdex 75 10/300 GL column of His_6_‐tagged wild‐type MthCTD (WT) and a variant with D611A and D613A substitutions of the predicted metal‐ion‐binding residues (DADA). The arrows indicate elution volumes of reference proteins. The inset shows the peak fraction of each preparation analyzed by SDS‐PAGE and Coomassie Blue staining. The expected molecular mass of MthCTD is 30.4 kDa. (b) Stability of the MthComEC CTD in the presence of different divalent metal cations as assessed by thermal shift assay. The CTD proteins possessed a His_6_ tag except where indicated (No His‐tag). The metal ions were added as 1 mM of the chloride salt with the exception of Zn^2+^ where the sulfate salt was used. Error bars represent the standard deviation, *denotes a statistically significant difference (*p* < .05) compared with the buffer condition in a Dunnett’s test, and # denotes a statistically significant difference (*p* < .05) between the WT and DADA variant in a Welch’s *t*‐test, *n* = 3. (c) Stabilization of the His_6_‐tagged MthComEC CTD by 1 mM Mn^2+^ ions as assessed by differential scanning calorimetry

We screened the CTDs of ComEC from 11 different organisms for soluble heterologous expression in *Escherichia coli*. Following this initial screening we were able to successfully overproduce and purify the ComEC CTD of the thermophilic Gram‐positive bacterium *Moorella thermoacetica* DSM 521 (hereafter termed MthCTD). A variant CTD (MthCTD‐DADA) was also produced in which the two potential metal‐binding aspartate residues in motif II (positions 611 and 613 in the full‐length protein) were substituted with alanine residues (Figure 1a). The purified MthCTD and MthCTD‐DADA proteins were monodispersed and monomeric as judged by size exclusion chromatography (Figure 2a).

The ComEC CTD is predicted to bind metal ions. To identify the metal ions involved, we took advantage of the fact that the bound metal ions are likely to stabilize the protein fold. Thermal shift assays were used to measure the stability of the CTD to unfolding in the presence of different metal ions. Initially, the assays were carried out with protein that retained a His_6_‐tag used for purification. Of the divalent metal cations tested, only Mn^2+^ gave a statistically significant increase in the melting temperature (T_m_) of MthCTD (by 13°C) (Figure 2b). By contrast, the MthCTD‐DADA variant was not stabilized by Mn^2+^, indicating that D611 and D613 are involved in binding this metal ion. Note that it is unlikely that these substitutions disrupt the overall fold of MthCTD because in the absence of added metal ions the substituted variant has a similar T_m_ to that of the wild type protein. Although the CTD of *B*. *subtilis* ComEC has been proposed to bind Zn^2+^ (Baker et al., 2016) neither MthCTD nor MthCTD‐DADA were stabilized by Zn^2+^. The effects of Co^2+^, Ni^2+^, and Cu^2+^ on CTD stability could not be tested because these metal ions interfered with the assay.

To exclude the possibility that the observed Mn^2+^‐dependent increase in MthCTD stability was mediated by metal ion binding to the His‐tag present on the recombinant CTD protein, the thermal shift assays were repeated on samples from which the tag had been removed. The untagged CTD was more thermostable than the tagged protein in the absence of metal ions (T_m_ of 63°C vs 55°C) (Figure 2b). Nevertheless, the addition of Mn^2+^, but not Zn^2+^, still increased the stability of the protein (T_m_ increase of 5°C) (Figure 2b). Again, removal of the motif II aspartate residues blocked Mn^2+^‐dependent stabilization of the untagged CTD. Thus, the Mn^2+^‐dependent stabilization of the CTD requires the motif II aspartate residues but not the His_6_‐tag.

Differential scanning calorimetry was used to corroborate the results of the thermal shift assays. For the His_6_‐tagged CTD, a 14°C increase in unfolding temperature was observed in the presence of Mn^2+^ (Figure 2c), which is in agreement with the 13°C increase in T_m_ measured in the thermal shift assay. The His_6_‐tagged MthCTD‐DADA variant had melting points at 44°C and 59°C in the absence of Mn^2+^, the higher of which corresponds well both to the T_m_ measured for this protein in the thermal shift assay and to the melting point of the wild‐type MthCTD domain when Mn^2+^ is not present (Figure 2c). This higher melting point was not altered by addition of Mn^2+^. By contrast, the lower melting point, which was not observed in the thermal shift assay, disappeared in the presence of Mn^2+^. This indicates that the MthCTD‐DADA variant retains some type of interaction with Mn^2+^ but that this is distinct from the Mn^2+^‐dependent stabilization of the wild‐type protein mediated by the aspartate residues.

Taken together, the stability assays suggest that MthCTD binds only Mn^2+^ out of the metal ions tested and that this binding requires the motif II aspartate residues.

### 2.2 Enzymatic activities of the ComEC C‐terminal domain

**FIGURE 3:**
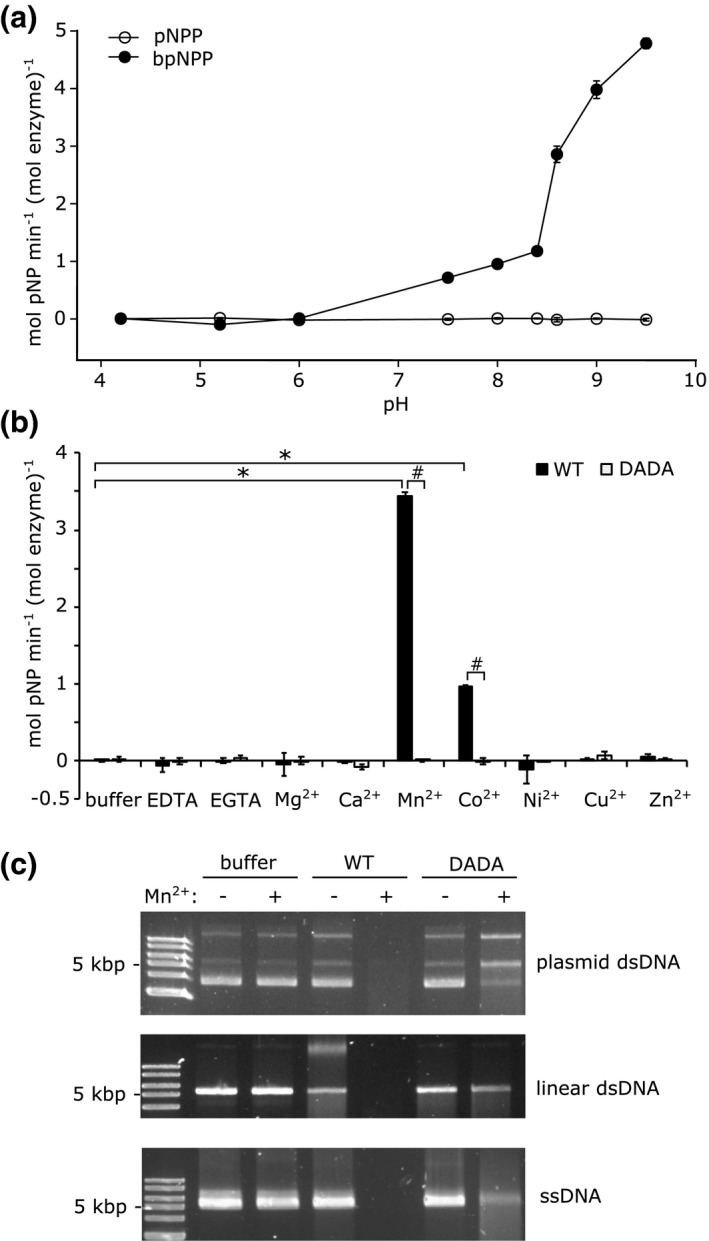
The ComEC CTD has phosphodiesterase and nuclease activities. (a) pH dependence of the phosphomonoesterase (with pNPP as substrate) and phosphodiesterase (with bpNPP as substrate) activities of the His_6_‐tagged MthComEC CTD in the presence of 1 mM Mn^2+^. The error bars show the standard deviation of three technical repeats. (b) Metal ion dependence of the phosphodiesterase activity of His_6_‐tagged MthComEC CTD at pH 9.0 with bpNPP as substrate. The metal ions were added as 1 mM of the chloride salt. The activities of both the wild‐type protein (WT) and of a variant (DADA) with D611A and D613A substitutions of the predicted metal‐ion‐binding residues are compared. The error bars show the standard deviation (three technical repeats). *denotes a statistically significant difference (*p* < .05) compared with the buffer condition in a Dunnett’s test, and # denotes a statistically significant difference (*p* < .05) between the WT and DADA variant in a Welch’s *t*‐test, *n* = 3. (c) The MthComEC CTD has nuclease activity. Supercoiled double‐stranded plasmid DNA, linearized plasmid DNA, or closed circle M13 phage single‐stranded DNA, were incubated at 50°C for 30 min either with no protein additions (buffer), or with 10 μM of either the WT or DADA variant of the His_6_‐tagged MthCTD. Where indicated the samples were supplemented with 5 mM MnCl_2_. The samples were subject to electrophoresis in an agarose gel and DNA detected with SYBR Gold stain. The experiment was repeated three times with similar results. The high‐molecular‐weight band in the condition where linearized plasmid DNA is incubated with WT protein in the absence of Mn^2+^ likely represents protein‐bound but uncleaved DNA

The structural homology of ComEC CTD to the MBL family suggests that it has hydrolytic activity, most likely acting as a DNA‐specific phosphodiesterase (Baker et al., 2016). Consistent with this hypothesis, we found that the purified MthCTD exhibited phosphodiesterase activity in the presence of Mn^2+^ with the synthetic phosphodiester substrate bis‐(*p‐*nitrophenyl) phosphate (bpNPP) (Figure 3a). Within the pH range 4.2–9.5, this activity was highest at pH = 9.5. Measurements at higher pH values were not possible due to precipitation of the Mn^2+^ ions. MthCTD exhibited negligible phosphomonoesterase activity against the phosphate monoester *p*‐nitrophenyl phosphate (pNPP) across the same pH range (Figure 3a). The absence of a phosphomonoesterase activity is consistent with the proposed nuclease role of the CTD because a phosphodiesterase activity is necessary for DNA degradation. The absence of phosphomonoesterase activity also demonstrates that the observed phosphodiesterase activity of the CTD is a specific catalytic reaction rather than arising from a generalized nonspecific hydrolytic activity of the domain.

The metal ion specificity of the observed phosphodiesterase activity of the MthCTD was assessed with a range of divalent metal cations, including some that could not be used in the earlier thermal shift stability assays. Significant phosphodiesterase activity was only observed in the presence of Mn^2+^ or Co^2+^ (Figure 3b). This activity was dependent on the metal‐binding aspartates in motif II of the CTD because the MthCTD‐DADA variant was catalytically inactive under the same conditions. Since the phosphodiesterase activity of MthCTD was higher with Mn^2+^ than with Co^2+^, and because the use of non‐corrin Co^2+^ is extremely rare in biology (Cracan & Banerjee, 2013), it is likely that Mn^2+^ is the physiologically relevant catalytic cofactor of ComEC.

To directly test the hypothesis that the ComEC CTD is a nuclease, we assessed the ability of purified MthCTD and MthCTD‐DADA to degrade DNA. For these experiments the CTD proteins were purified without addition of DNase to the lysis buffer to minimize contaminating nuclease activity. MthCTD was able to degrade supercoiled plasmid DNA, linear double‐stranded DNA, and single‐stranded DNA when assayed at a temperature (50°C) approximating the *M*. *thermoacetica* growth optimum (Figure 3c). The CTD had this nuclease activity in the presence of Mn^2+^ ions but not without added metal ions with the exception of trace activity with linear double‐stranded DNA. The nuclease activity was also dependent on the metal‐ion–binding aspartate residues since MthCTD‐DADA had only minor nuclease activity compared with the WT variant in the presence of Mn^2+^ ions (we cannot eliminate the possibility that even this residual activity is due to trace nuclease contamination from the *E. coli* expression host). These data show that MthCTD is a Mn^2+^‐dependent DNA nuclease. Because the CTD is able to degrade circular double‐stranded plasmid DNA, it must be able to function as an endonuclease.

### 2.3 The nuclease activity of the ComEC CTD is important for natural transformation

The experiments above show that MthCTD has a metal‐dependent nuclease activity. To examine the importance of this nuclease activity in natural transformation, we constructed a strain of *B*. *subtilis* that produces a variant ComEC (ComEC DADA) in which the metal‐binding aspartate residues of the CTD motif II are replaced with alanine residues (the *B*. *subtilis* ComEC CTD retains all the predicted metal ion ligands found in the *M*. *thermoacetica* protein and the two protein domains are 31% identical in amino acid sequence). By analogy to the in vitro experiments with the equivalent MthCTD variant, these substitutions are expected to abolish the nuclease activity of the CTD. The ComEC protein was also modified by addition of an epitope tag to allow immunological detection.

**FIGURE 4:**
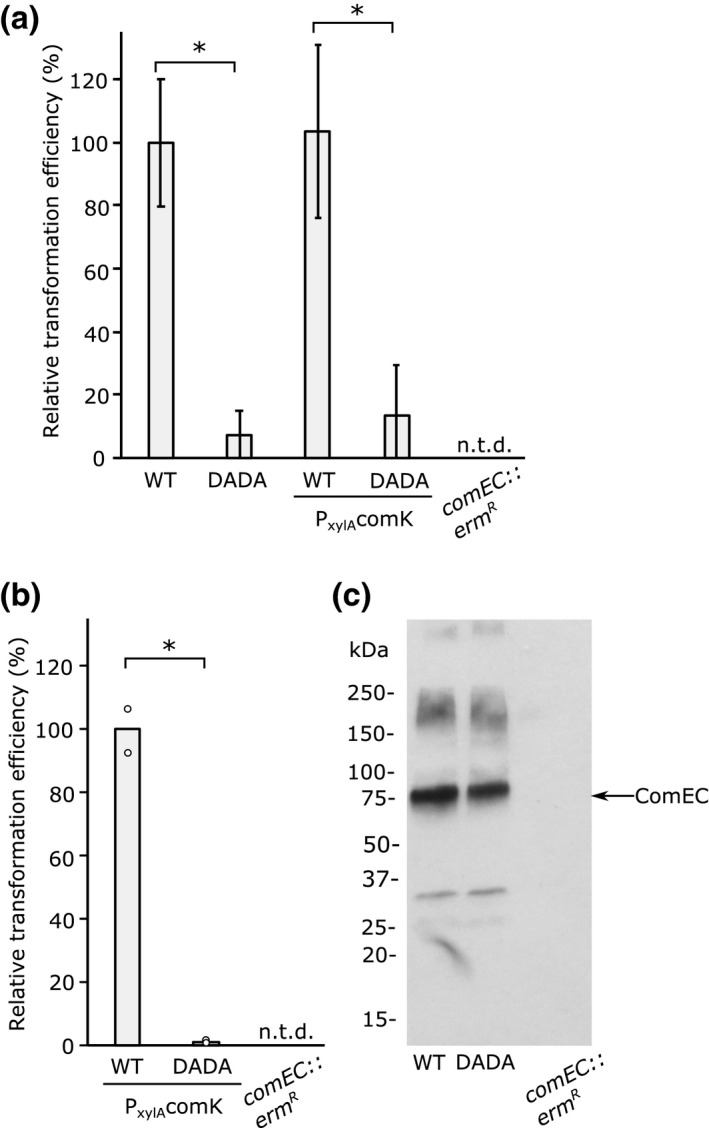
ComEC nuclease activity is required for efficient transformation. (a,b) Comparison of the transformation efficiency of *B. subtilis* strains encoding either a wild‐type (WT) *comEC‐twinstrep* gene or a metal‐ion‐binding *comEC‐twinstrep D573A*, *D575A* mutant (DADA). In some strains, the background also contains a P*_xylA_comK* allele, which directs expression of the competence regulator ComK under the control of a xylose‐inducible promoter. Cells were transformed with genomic DNA carrying a chloramphenicol resistance gene. A *comEC* null mutant (*comEC::erm^R^)* served as a negative control for transformation. N.t.d., no transformants detected. The error bars show the standard deviation of the values (*n* = 3 (a) or *n* = 2 (b) biological repeats for each strain). *indicates a significant difference (*p* < .05) between values in a Welch’s *t*‐test. The transformation efficiency of the WT cells was 1.6 × 10^−5^ ± 0.3 × 10^−5^ (S.D.) in (a) and 1.6 × 10^−2^ ± 0.1 × 10^−2^ in (b). (a) Competence was induced by nutrient starvation. Under these conditions, only a small proportion of the cells in the population are competent for DNA uptake. (b) Competence was induced in the whole cell population through xylose induction of the P*_xylA_comK* allele during growth on a rich medium. (c) The relative expression levels of the WT and DADA variant ComEC proteins induced as in (b) and assessed by immunoblotting the total membrane fraction with Streptag antibodies

Competence was induced either by nutrient limitation or by overproduction of the competence regulator ComK from a xylose‐regulated promoter during growth on a rich medium. In both cases, the strain expressing the ComEC DADA variant could still be transformed with genomic DNA (Figure 4a,b). However, the transformation efficiency of the mutant was approximately 10‐fold lower than that of the parental strain when competence was induced by nutrient limitation (Figure 4a) and approximately 100‐fold lower than that of the parental strain when competence was induced by ComK overproduction (Figure 4b), the difference presumably reflecting the degree to which ComEC is the limiting step in transformation under the two competence conditions. No transformation was detected for a strain in which the *comEC* gene was inactivated confirming that ComEC is essential for DNA uptake under our experimental conditions (Figure 4a,b).

To exclude the possibility that the aspartate substitutions in the DADA variant affect the biogenesis or stability of the ComEC protein, immunoblotting was used to quantify ComEC levels. This analysis confirmed that the ComEC DADA variant was present in the cells at the same levels as the parental protein when competence was induced by ComK overproduction (Figure 4c; our immunoblotting assay was insufficiently sensitive to detect the lower levels of ComEC induced by nutrient limitation).

In conclusion, our data show that the nuclease activity of the ComEC CTD is important for natural transformation in *B*. *subtilis*.

## 3 DISCUSSION

The ComEC protein of the canonical competence system of *B*. *subtilis* contains an extracellular MBL‐like domain (the CTD). An analogous domain is present in approximately half of the ComEC proteins encoded in bacterial genomes (Pimentel & Zhang, 2018). The function of this domain has never been investigated, but it has been suggested to be a metal‐dependent nuclease involved in degrading the nontransforming DNA strand during DNA uptake across the cell envelope (Baker et al., 2016; Dubnau & Blokesch, 2019). Here we have investigated the biochemical properties of the CTD and its role in natural transformation.

The ComEC CTD has been predicted to bind two Zn^2+^ ions based on the known metal content of the most closely related MBL fold proteins (Baker et al., 2016). However, our biochemical analysis of the isolated CTD of the ComEC protein of *M*. *thermoacetica* indicates that the domain binds Mn^2+^ rather than Zn^2+^ or other metal ions. This conclusion is based both on the thermodynamic stabilization of the domain by Mn^2+^ and by the ability of Mn^2+^ to support the enzymatic activity of the domain. The inferred protein ligands of the Mn^2+^ ions are three histidine residues, three terminal and one bridging aspartate residues, and one asparagine residue. These ligands are consistent with the O‐ and N‐ co‐ordination expected of protein‐bound Mn^2+^ ions (Christianson, 1997) and fall within the range of co‐ordinating side chain atoms found in structurally characterized dinuclear Mn^2+^ binding proteins (e.g., the thiosulfohydrolase SoxB with 5 × N, 3 × O (Sauve et al., 2009) and arginase with 2 × N, 6 × O (Kanyo et al., 1996)). The metal ion ligands are conserved in most other ComEC CTDs, including that found in the *B*. *subtilis* ComEC, leading us to expect that Mn^2+^ is the metal ion that is normally bound to this domain.

The enzyme responsible for digesting the nontransforming DNA strand would be anticipated to be an exonuclease because it is expected to act processively on the transporting DNA molecule and also because in *B*. *subtilis* the incoming DNA molecules are already linearized by the endonuclease NucA (Provvedi et al., 2001). Nevertheless, we find that the isolated ComEC CTD is able to digest circular double‐ and single‐stranded DNA molecules (Figure 3c) and so must also possess some endonuclease ability. In our in vitro experiments, the ComEC CTD was ultimately able to digest both strands of double‐stranded DNA molecules (Figure 3c). Presumably, digestion of the transforming strand is prevented in the physiological context because it is transported immediately after it leaves the CTD active site.

*B. subtilis* takes up DNA during natural transformation at a rate of 80 bp/s (Maier et al., 2004). Assuming that the rate of degradation of the nontransforming strand is similar to the rate of DNA uptake in *B*. *subtilis*, as is the case in *S*. *pneumoniae* (Mejean & Claverys, 1993), then the rate of DNA hydrolysis by the CTD should be ~80 phosphodiester bonds per second. The rate of bpNPP hydrolysis by MthCTD in the presence of Mn^2+^ was 3.4 mol/min (mol protein)^−1^ (Figure 3b), which corresponds to ~0.06 molecules of bpNPP molecules hydrolyzed per second per molecule of MthCTD. Thus, the rate of bpNPP hydrolysis by MthCTD is three orders of magnitude lower than that would be required for degradation of the nontransforming strand in *B*. *subtilis*. A number of factors could contribute to this discrepancy. First, the test substrate bpNPP does not closely resemble a DNA molecule. Second, the activity of the CTD may be affected by being taken out of its native context. For example, other domains of ComEC, or other components of the transformation machinery, may orient or tension the DNA molecule appropriately for hydrolysis and may be required to actively move the strand through the ComEC active site. Third, the CTD tested comes from an organism that grows optimally at 55°C, but for technical reasons, the phosphodiesterase activity of the isolated CTD was assayed at 30°C. Thus, the measured phosphodiesterase activity is likely to significantly underestimate the activity of the enzyme under physiological conditions.

Our data show that the metal‐dependent nuclease activity of the ComEC CTD is important for transformation in *B*. *subtilis* since the efficiency of transformation dropped between 10‐ and 100‐fold (depending on the experimental system) when the CTD could no longer bind metal ions (a D573A, D575A variant) but did not block transformation completely (Figure 4a,b). However, we note that a contrary recent study reports that substitution of one of the predicted metal‐binding residues replaced here (D573) completely prevents transformation in *B*. *subtilis* (bioRxiv preprint https://doi.org/10.1101/2020.09.29.319830; individual substitutions of other predicted metal ion ligands had partial effects on transformation in that study). The reason for the discrepancy in phenotypes between the two studies is not clear. For comparison, deleting the EndA nuclease required for digestion of the nontransforming strand in *S*. *pneumoniae* reduces transformation efficiency around ~100‐fold, but again it does not completely abolish the process (Berge et al., 2002; Puyet et al., 1990).

The residual transformation activity that we observe in the *B*. *subtilis comEC* DADA mutant may be due to the CTD retaining trace nuclease activity as suggested by our characterization of the equivalent MthComEC CTD variant (Figure 3c). Alternatively, we speculate that a small region of single‐stranded DNA is formed at the end of the transforming DNA, either through transient DNA unwinding or through the action of a nonspecific extracellular nuclease, and that this end of the DNA molecule is then trapped by the OBD domain or catalytically inactive CTD. We propose that this enables translocation of end of the DNA molecule through the ComEC pore. Once the single‐stranded end of the DNA has reached the cytoplasm, we envisage that the pulling action of the ComFA motor protein is strong enough to force a slow unwinding of the bulk of the DNA molecule at the extracellular face of the membrane as ComFA reels the transforming strand through the transport pore.

In *S*. *pneumoniae* the nontransforming strand of the incoming DNA is degraded by the nuclease EndA. Nevertheless, the ComEC protein of *S*. *pneumoniae* still contains an MBL‐like CTD. This raises the questions as to why EndA is required by the organism and why the CTD is retained. The *S*. *pneumoniae* CTD has several substitutions of the metal‐binding residues relative to other ComEC CTDs: Asn for His and Glu for Asp in motif II (Figure 1b), and the metal bridging Asp from motif IV is replaced by Asn (not shown in Figure 1b). It is not clear that these relatively conservative sequence changes would prevent the domain‐binding metal ions or having nuclease activity. However, if the *S*. *pneumoniae* CTD is catalytically inactive, it may be retained to act as a guide for the transported DNA molecule.

Almost half the ComEC proteins encoded in sequenced genomes lack a CTD of the type characterized here (Pimentel & Zhang, 2018). How the nontransforming DNA strand is degraded (or perhaps displaced) in these organisms remains an open and interesting question.

## 4 EXPERIMENTAL PROCEDURES

### 4.1 Bacterial strains and growth conditions

All strains and plasmids used in this work are listed in Tables 1 and 2. *E. coli* TOP10 cells were used for routine cloning work, and strain MC1061 was used to propagate plasmids prior to transformation of *B*. *subtilis*. *E. coli* and *B*. *subtilis* strains were routinely grown in Luria Bertani (LB) medium (Bertani, 1951) at 37°C with shaking. When required, antibiotics were added at the following concentrations: ampicillin, 100 µg ml^−1^, kanamycin, 50 μg ml^−1^, spectinomycin, 100 μg ml^−1^, chloramphenicol, 25 μg ml^−1^ for *E. coli* and 5 μg ml^−1^ for *B*. *subtilis*, erythromycin, 1 or 5 μg ml^−1^, lincomycin, 25 μg ml^−1^.

**TABLE 1:** Strains used in this study

Strain	Description	Donor strain/plasmid	Reference
*Escherichia coli*			
MC1061	*araD139* Δ(*araA‐leu)7697* Δ(*lac*)*X74 galK16 galE15*(*GalS*) *λ^−^ e14^−^ mcrA0 relA1* r*psL150* (*Str^R^*) *spoT1 mcrB1 hsdR2*		Casadaban and Cohen (1980)
TOP10	MC1061 *Δ(mrr‐hsdRMS‐mcrBC) φ80lacZΔM15 nupG recA1 endA1*		Invitrogen
BL21(DE3) pLysS	*ompT hsdS*_B_(r_B_^−^ m_B_^−^) *gal* *dcm* (DE3) pLysS (Cam^R^)		Invitrogen
*B. subtilis*			
168+	Wild type strain with restored tryptophan prototrophy, *trpC2^+^*		R.A. Daniel (Newcastle)
1A976	*his nprE18 aprE3 eglSΔ102 bglT/bglSΔEV lacA::P_xylA_‐comK (erm^R^)*		Zhang and Zhang (2011)
BKE25570	*trpC2 comEC::erm^R^*		Koo et al. (2017)
AS7	168+ *comEC‐twinstrep*	pMAD‐ComEC‐TS	This study
AS7K	168+ *comEC‐twinstrep lacA::P_xylA_comK (erm^R^)*	1A976	This study
AS8	168+ *comEC^(DADA)^‐twinstrep*	pMAD‐ComEC‐DADA	This study
AS8K	168+ *comEC^(DADA)^‐twinstrep lacA::P_xylA_comK (erm^R^)*	1A976	This study
AS14	168+ *comEC‐twinstrep comEC::erm^R^*	BKE25570	This study
AS20	168+ *amyE::P_comGA_‐lacZ‐gfp (cam^R^) lacA::P_xylA_comK (erm^R^)*	pPG40, 1A976	This study

**TABLE 2:** Plasmids used in this study

Plasmid	Description	Reference
pGEM T Easy	TA cloning	Promega
pCDFDuet‐1	Expression from dual *lac‐pT7* promoters *ori pCloDF13 lacI spc^R^/str^R^*	Novagen
pET‐28b(+)	Expression from *lac‐pT7* promoter *ori pBR322 kan^R^*	EMD Biosciences
pMAD	Used for markerless allelic exchange in *Bacillus* *subtilis*, *ori pE194^ts^ ori pBR322 bgaB bla ermC*	(Arnaud et al., 2004)
pPG40	*amyE3′ (bla) P_comG_‐lacZ‐gfp+ (cam^R^) amyE5′*	(Gamba et al., 2015)
pCDFDuet‐MthCTD	pCDFDuet‐1 derivative. Expression of His_6_‐MthComEC(541‐809)	This study
pCDFDuet‐MthCTD‐DADA	pCDFDuet‐1 derivative. Expression of His_6_‐MthComEC(541‐809)^(D611A, D613A)^	This study
pCDFDuet‐TEV‐MthCTD	pCDFDuet‐1 derivative. Expression of His_6_‐TEV‐MthComEC(541‐809)	This study
pCDFDuet‐TEV‐MthCTD‐DADA	pCDFDuet‐1 derivative. Expression of His_6_‐TEV‐MthComEC(541‐809)^(D611A, D613A)^	This study
pMAD‐ComEC‐TS	pMAD derivative. Construction of *comEC‐twinstrep*	This study
pMAD‐ComEC‐DADA	pMAD derivative. Construction of *comEC^(DADA)^‐twinstrep* mutant	This study

### 4.2 Construction of expression plasmids

All primers used in this work are listed in Table S1. All constructs were verified by sequencing.

A plasmid to heterologously express the *M*. *thermoacetica* ComEC CTD with a N‐terminal His_6_ tag (MGSSHHHHHHS‐) was constructed as follows. A fragment of the *M*. *thermoacetica comEC* gene covering codons 541‐809 was amplified from genomic DNA with primers AS31 and AS32 and inserted by In‐Fusion cloning (Takara Bio) into pCDFDuet‐1 (Novagen) that had been linearized by amplification with primers AS33 and AS34 to yield plasmid pCDFDuet‐MthCTD. The Q5 site‐directed mutagenesis kit (NEB) was used to introduce D611A and D613A substitutions into the CTD‐coding region with primers AS35 and AS36, resulting in plasmid pCDFDuet‐MthCTD‐DADA. The pCDFDuet‐MthCTD and pCDFDuet‐MthCTD‐DADA constructs were further modified to introduce a TEV cleavage site between the His_6_‐tag and the CTD using the Q5 site‐directed mutagenesis kit and primers AS37 and AS38, producing plasmids pCDFDuet‐TEV‐MthCTD and pCDFDuet‐TEV‐MthCTD‐DADA.

### 4.3 Purification of the *M*. *thermoacetica* ComEC CTD

BL21 (DE3) pLysS cells transformed with pCDFDuet‐MthCTD, pCDFDuet‐TEV‐MthCTD, pCDFDuet‐MthCTD‐DADA, or pCDFDuet‐TEV‐MthCTD‐DADA plasmids were grown in Terrific Broth (Tarto & Hobbs, 1987) with chloramphenicol and spectinomycin at 37°C with 180 rpm shaking. When cultures reached OD_600_ = 0.6–0.8 expression was induced by adding 0.5 mM IPTG. The cells were grown for a further 4 hr at 30°C. Cells were harvested by centrifugation at 6,000 ×*g* for 30 min at 4°C. Cell pellets were flash‐frozen in liquid nitrogen and stored at −80°C until use.

Cell pellets were resuspended in buffer A (50 mM Tris‐HCl pH 8.0, 300 mM NaCl) supplemented with a few crystals of lysozyme and 1 mM phenylmethylsulfonyl fluoride (PMSF) (Sigma) and incubated with stirring at room temperature for 30 min. The suspension was homogenized, and cells were lysed by sonication in a 130 W Vibra‐Cell sonicator at 60% amplitude for 10 min on ice. The lysate was clarified by centrifugation at 30,000 ×*g* for 30 min at 4°C. The supernatant was passed through a 0.45‐μm filter, supplemented with imidazole to a final concentration of 20 mM, and incubated with NiNTA agarose beads (QIAGEN) at 4°C for 18 hr with gentle stirring. Beads with bound protein were collected on a gravity flow column, washed with 30 CV buffer A with 20 mM imidazole, 30 CV buffer A with 30 mM imidazole, and eluted with 5 CV buffer A with 125 mM imidazole. The eluate was immediately diluted twofold with buffer A and stored at 4°C. The diluted eluate was concentrated in a spin concentrator with a 10 kDa cutoff membrane (Amicon Ultra, Merck) and subjected to size exclusion chromatography on a Superdex 75 10/300 or HiLoad Superdex 75 10/60 prep‐grade column (GE) equilibrated in 20 mM Tris‐HCl pH 8.0, 150 mM NaCl. Fractions containing pure protein were identified by SDS‐PAGE.

Constructs containing a TEV protease cleavage site were incubated with His_6_‐tagged TEV protease (1:100 protease to target protein mass ratio) after the NiNTA affinity purification step and dialyzed against buffer A for 16 hr at 4°C. The samples were then incubated with NiNTA beads to remove the cleaved His_6_‐tag, the protease and any uncleaved fusion protein, and the flow‐through was concentrated and subjected to size exclusion chromatography as above.

### 4.4 Biophysical methods

Thermal shift assays for metal binding were carried out in 96‐well format in a Stratagene MX3005P real‐rime PCR machine. 25 μl reactions contained 12 μM ComEC CTD, 1 mM of the metal salt under investigation, 1:5,000 diluted SYPRO Orange dye (Invitrogen), 18 mM Tris‐HCl pH 8.0, and 135 mM NaCl. Samples were heated from 25°C to 94°C in 1°C increments, allowing 30 s for equilibration at each set temperature before fluorescence readings were taken. The melting temperature of the protein (T_m_) was extracted by using TSA‐CRAFT software (Lee et al, 2019) to fit the data to the Boltzmann equation.

Differential scanning calorimetry measurements were performed on a Malvern VP Capillary DSC instrument. The sample cell contained 18.6 μM ComEC CTD protein in 20 mM Tris‐HCl, 150 mM NaCl and, where appropriate, 1 mM MnCl_2_. The temperature was raised from 10°C to 110°C at a rate of 200°C/h. Runs without protein were used for baseline subtraction. Data were analyzed using the instrument software. The baseline was subtracted from the raw data, and the data were normalized to protein concentration. The data were fitted to a two‐state unfolding model to extract the T_m_.

### 4.5 Phosphatase activity assays

pNPP or bpNPP (both from Sigma) were used as the substrates for, respectively, phosphomonoesterase and phosphodiesterase activity assays (Rodriguez et al, 2014). Reactions (100 μl) were performed in 96‐well format in a CLARIOstar Plus plate reader (BMG Labtech) at 30°C in 40 mM glycine‐NaOH pH 9.5, 60 mM NaCl, 4 mM MnCl_2_, and at a final enzyme concentration of 1 μM. Reactions were started by addition of the substrate to a final concentration of 0.8 mM and monitored at 405 nm for 10 min. A linear regression fit was performed in MARS Data Analysis software (BMG Labtech) to extract the slopes from the absorbance versus time curves and the amount of *p*‐nitrophenol released was calculated using ε_405_(*p*‐nitrophenol) = 18,000 M^−1^ cm^−1^.

### 4.6 Nuclease assays

Ten μM test protein was incubated with 25 ng double‐stranded supercoiled plasmid DNA (pET28b, EMD Biosciences), NdeI‐linearized double‐stranded plasmid DNA (pET28b), or M13mp18 phage single‐stranded DNA (NEB) at 50°C in 50 mM glycine‐NaOH pH 9.0, 75 mM NaCl, supplemented with 5 mM MnCl_2_ as indicated. After 30 min reactions were quenched by adding 6× purple loading dye (NEB) with 5 mM EDTA and incubation on ice. Samples were analyzed by agarose gel electrophoresis. Gels were stained with SYBR Gold (ThermoFisher).

### 4.7 Transformation of *Bacillus subtilis*

Induction of competence by nutrient limitation was based on the protocol of Bennallack et al (2014). Overnight LB cultures were used to inoculate 15 ml of prewarmed SPC medium (1 mM MgSO_4_, 0.5% (w/v) glucose, 0.2% (w/v) yeast extract, 0.025% (w/v) casamino acids, 0.2 g/L (NH_4_)_2_SO_4_, 1.83 g/L K_2_HPO_4_, 0.6 g/L KH_2_PO_4_, 0.1 g/L trisodium citrate dihydrate; modified from (Spizizen, 1958)) in a 250 ml flask to a starting OD_600_ = 0.3–0.4. Cultures were grown at 37°C with 180 rpm until stationary phase (OD_600_ = ~1.8; 3–4 hr). The cultures were then diluted by adding an equal volume of prewarmed SPII medium (as SPC, but with 3.5 mM MgSO_4_, 1 mM CaCl_2_, 0.1% yeast extract, 0.01% casamino acids) and grown for another 1.5 hr until competence.

Induction of competence through induction of P*_xylA_comK* used the protocol of (Zhang & Zhang, 2011). An overnight LB culture was diluted to OD_600_ = 1.0 in LB with 1% xylose and grown for 2 hr at 37°C, shaking at 180 rpm, to allow expression of competence genes.

Genomic DNA for the transformation assays was prepared from *B*. *subtilis* AS20 using a PureLink Genomic DNA Mini Kit (Thermo Fisher Scientific). DNA concentration was estimated by measuring absorbance at 260 nm. One μg of genomic DNA was added to 500 μl competent cells in a sterile glass test tube. Cells were incubated with DNA for 30 min at 37°C with 180 rpm shaking. 300 μl of LB was then added, and the cells were incubated for a further 30 min before plating. The transformation efficiency is defined as the ratio of the number of transformants observed on selective medium (LB‐chloramphenicol agar) to the number of colony‐forming units observed on nonselective medium (LB).

### 4.8 Construction of unmarked *B*. *subtilis* chromosomal integrants

The pMAD suicide plasmid system (Arnaud et al., 2004) was used to construct unmarked chromosomal alterations in *B*. *subtilis*. All plasmid insert sequences were confirmed by sequencing.

A plasmid for appending a C‐terminal TwinStrep tag to the ComEC protein (strain AS7) was constructed as follows. A 1,900 bp fragment incorporating the *comEC* gene was amplified from *B*. *subtilis* subsp. *subtilis* 168 genomic DNA using primers AS39 and AS40 and ligated into pGEM‐T Easy (Promega) using TA cloning. The resulting plasmid was then linearized by amplification with primers AS41 and AS42 and then ligated using In‐Fusion cloning to a fragment encoding the TwinStrep tag (WSHPQFEKGGGSGGGSGGSAWSHPQFEK) amplified with primers AS43 and AS44 from a plasmid carrying the TwinStrep coding sequence. The fragment containing the tagged coding sequence was then amplified using primers AS45 and AS46 and ligated using the In‐Fusion reaction into BamHI‐ and SalI‐digested pMAD (Arnaud et al., 2004) yielding plasmid pMAD‐ComEC‐TS.

A plasmid for removing the putative metal‐binding ligands from the CTD of ComEC (strain AS8) was constructed as follows. A 1,200 bp fragment including the *comEC* gene was amplified from *B*. *subtilis* subsp. *subtilis* 168 genomic DNA using primers AS61 and AS62 and ligated into pGEM‐T Easy by TA cloning. Nucleotides coding for ComEC residues D573 and D575 were mutated to alanine‐coding nucleotides using the Q5 site‐directed mutagenesis kit with primers AS63 and AS64. The fragment containing the mutated codons was then amplified using primers AS65 and AS66 and cloned into pMAD as above to yield plasmid pMAD‐ComEC‐DADA.

*B. subtilis* cells were grown to competence under starvation conditions as described above. 3 μg of the appropriate pMAD plasmid were added to 500 μl competent cells in glass test tubes and incubated for 1 hr at 30°C with 180 rpm shaking. Cells were plated on LB agar plates containing MLS (25 μg/ml lincomycin and 1 μg/ml erythromycin) and incubated at 30°C for 2 days. Two colonies were picked into separate 5 ml LB‐MLS cultures and incubated overnight at 37°C with 180 rpm shaking to promote integration of the temperature‐sensitive plasmid. The following morning the culture was serially diluted 10^−4^ to 10^−6^, plated on LB‐MLS agar plates containing 100 μg/ml 5‐bromo‐4‐chloro‐3‐indolyl‐β‐D‐galactopyranoside (X‐gal), and then incubated overnight at 37°C. The following day, four blue colonies were picked and used to inoculate separate 5 ml LB cultures. These cells were then cultured at 30°C overnight without shaking to promote excision of the pMAD plasmid from the chromosome. The following morning, the cultures were incubated for a further 4 hr at 30°C with 180 rpm shaking. The cultures were serially diluted 10^−6^ to 10^−8^ and plated on LB‐X‐gal agar plates, which were incubated at 37°C overnight. The following day, white colonies were replica‐patched on LB‐X‐gal and LB‐MLS agar plates followed by overnight incubation at 37°C. Patched colonies that were white and MLS‐sensitive were screened by PCR and sequencing to confirm the desired alterations to the chromosome. Strains were stored as glycerol stocks at −80°C.

### 4.9 Isolation of membrane fractions and Western blotting

Strains were cultured and induced for competence as for the transformation assays above. Cell pellets were harvested and stored at −20°C. Frozen pellets were resuspended in 2 ml ice‐cold phosphate‐buffered saline (Sigma) with 1 mM EDTA (PBSE), lysozyme and DNase I, and incubated on ice for 30 min. Cells were lysed by sonication using a 130 W Vibra‐Cell sonicator at 100% amplitude for 1 min on ice. Cellular debris and unbroken cells were removed by centrifugation at 15,000 ×*g* for 10 min at 4°C. The membrane fraction was pelleted by ultracentrifugation at 220,000 ×*g* for 1 hr, at 4°C. The membrane fraction was resuspended in 200 μl PBSE, and the total protein content was estimated using the DC Assay (Bio‐Rad). 90 μg of membrane protein from each strain was subjected to SDS‐PAGE and analyzed by immunoblotting with a mouse anti‐Strep‐tag II primary antibody (QIAGEN) (1:2,000 dilution) and a polyclonal goat anti‐mouse antibody coupled to horseradish peroxidase (Sigma) (1:10,000 dilution).

## AUTHOR CONTRIBUTIONS

A. Silale carried out all experiments. B.C. Berks and S.M. Lea conceived the project. All authors interpreted data and wrote the manuscript.

## Supporting information

Table S1

## Data Availability

The data that support the findings of this study are available from the corresponding authors on reasonable request.
