# Supplementary material for: The DNA transporter ComEC has metal‐dependent nuclease activity that is important for natural transformation
Source: Mol Microbiol. 2021 Jun 4;116(2):416–26. doi: 10.1111/mmi.14720 (PMC8579336; doi:10.1111/mmi.14720)
Supplement: Supplementary file 1 — Table S1 [file MMI-116-416-s001.docx]

**Supplementary Information**

**The DNA transporter ComEC has metal-dependent nuclease activity that is important for natural transformation**

Augustinas Silale^1,2,3^, Susan M. Lea^2,4†^, and Ben C. Berks^1†^

^1^ Department of Biochemistry, University of Oxford, South Parks Road, Oxford OX1 3QU, United Kingdom.

^2^ Sir William Dunn School of Pathology, University of Oxford, South Parks Road, Oxford OX1 3RE, United Kingdom.

^3^ Current address: Biosciences Institute, Newcastle University, Cookson Building, Newcastle upon Tyne NE2 4HH, United Kingdom.

^4^ Current address: Center for Structural Biology, Center for Cancer Research, National Cancer Institute, Building 538, Frederick MD 21702-1201, USA

† To whom correspondence should be addressed. S.M.Lea ([susan.lea@nih.gov](mailto:susan.lea@nih.gov)); B.C.Berks ([ben.berks@bioch.ox.ac.uk](mailto:ben.berks@bioch.ox.ac.uk)).

**SI Table 1 Primers used in this study.**

| Name | Sequence 5′-to-3′ |
| --- | --- |
| AS31 | ATCACCATCATCACCACAGCGGGCAGCCGGGAGAAC |
| AS32 | GAGCTCGAATTCGGATCCTGTTAAGGGAGAACAGTCTTTACCTGC |
| AS33 | CTAGTTCTCCCGGCTGCCCGCTGTGGTGATGATGGTGATG |
| AS34 | GTAAAGACTGTTCTCCCTTAACAGGATCCGAATTCGAGC |
| AS35 | TGCTCATCTGGGCGGCCTCATG |
| AS36 | GTAGCCGGGTGGGTGCTGACGAC |
| AS37 | TACTTCCAGGGGCAGCCGGGAGAACTA |
| AS38 | CAGGTTTTCGCTGTGGTGATGATGGTGATG |
| AS39 | ATAGTCGACACGATCTCCAATAAACG |
| AS40 | ATAGGATCCGTAACCGGAGGCATTTG |
| AS41 | GTTCGTCTCTGTTATATCTGATGT |
| AS42 | TAAAAAAGACTGCCGAGAAATCAG |
| AS43 | CGGCAGTCTTTTTTATTTCTCGAACTGCGGGTGG |
| AS44 | ATAACAGAGACGAACTGGAGCCATCCGCAGTTTG |
| AS45 | CTGCAGACGCGTCGAACGATCTCCAATAAACGTGCAGAGC |
| AS46 | GGGCGATATCGGATCGTAACCGGAGGCATTTGCTGC |
| AS61 | CGTCTCTGTTATATCTGATGTATC |
| AS62 | CAGTTTTCTATCATCAGCGTAC |
| AS63 | GCGCAGCGTGCGTCAGAATTAAAG |
| AS64 | AAGCGCATATCGGAGAGGCGGAG |
| AS65 | CTGCAGACGCGTCGACGTCTCTGTTATATCTGATGTATCA |
| AS66 | GGGCGATATCGGATCCAGTTTTCTATCATCAGCGTACCGA |
